# A self-efficacy-enhancing physical activity intervention in women with high-risk factors for gestational diabetes mellitus: study protocol for a randomized clinical trial

**DOI:** 10.1186/s13063-022-06379-6

**Published:** 2022-06-06

**Authors:** Xiao Yang, Ji Zhang, Xiangzhi Wang, Yi Xu, Li Sun, Yingli Song, Ruijuan Bai, Hui Huang, Jing Zhang, Ruixing Zhang, Erfeng Guo, Lingling Gao

**Affiliations:** 1 School of Nursing, Sun Yat-Sen University, No. 74 Zhongshan Road 2, Yuexiu District, Guangzhou, Guangdong Province 510080 P.R. China; 2 Women and Infants Hospital of Zhengzhou, Zhengzhou, China; 3 School of Nursing, Zhengzhou University, Zhengzhou, China

**Keywords:** Physical activity, Exercise, Gestational diabetes, Self-efficacy, Randomized controlled trial, Protocol

## Abstract

**Background:**

Gestational diabetes mellitus (GDM) is one of the most common medical disorders in pregnancy. Evidence has demonstrated that moderate-intensity physical activity may reduce the risk of gestational diabetes. However, women at risk of GDM spend most of their time performing sedentary behaviors. Although researchers identified self-efficacy as a mediator to overcome physical activity barriers, exercise intervention during pregnancy based on self-efficacy theory has not been discussed so far. Furthermore, there is conflicting evidence regarding the effects of a physical exercise intervention on the incidence of GDM and other maternal or neonatal outcomes in women at higher risk for GDM.

**Methods/design:**

A single-center, parallel, randomized controlled trial will be conducted in a maternal–child health care center. A total of 244 pregnant women at high risk for GDM will be randomized into a study group receiving a self-efficacy-enhancing physical activity intervention or a control group receiving the usual care. The intervention will consist of four group sessions and everyday reminders by WeChat (Tencent, Shenzhen, China). The program will begin at approximately 13–14^+6^ gestational weeks and end at 36^+6^ gestational weeks. The primary outcomes will include the incidence of GDM, blood sugar values, and physical activity. The secondary outcomes will include physical activity self-efficacy, gestational weight gain, maternal outcomes, and neonatal outcomes.

**Discussion:**

The findings of this research will contribute toward understanding the effects of a self-efficacy theory-oriented physical activity program on the incidence of GDM, blood sugar values, physical activity level, gestational weight gain, physical activity self-efficacy, maternal outcomes, and neonatal outcomes.

**Trial registration:**

Chinese Clinical Trial Registry (CHiCTR) ChiCTR2200056355. Registered on February 4, 2022.

## Background

Gestational diabetes mellitus (GDM) is one of the most common medical disorders in pregnancy [1] and is associated with a greater risk of both short- and long-term adverse health outcomes for mothers and their offspring [2–6]. The prevalence of GDM is increasing worldwide, with an incidence rate of 16.7% documented in 2021 [7]. 14.8% of all women in mainland China were diagnosed with GDM in 2020 [8]. Well-recognized risk factors for GDM include higher maternal age, higher pre-pregnancy body mass index (BMI), family history of type 2 diabetes, polycystic ovary syndrome, hypothyroidism, previous macrosomia, ethnicity, certain lifestyle factors, and GDM or glucose intolerance in previous pregnancies [9–11]. Women with a combination of risk factors will have an even greater increased risk of GDM. Research has revealed that GDM reoccurs in nearly half of women with a history of GDM [12]. Thus, the best strategy to prevent GDM is to target the high-risk population [13].

Evidence has demonstrated that regular moderate-intensity physical activity (PA) may reduce the risk of gestational diabetes [14]. Furthermore, women with regular PA during pregnancy were more likely to give birth vaginally and less likely to have preeclampsia, gestational hypertension, excessive gestational weight gain, postpartum weight retention, or postpartum depression [14–17]. Thirteen clinical practice guidelines consistently recommend that all healthy pregnant women perform 150 min of moderate PA per week [18]. However, researchers have found that PA in women decreases from pre-pregnancy to pregnancy [19]. The majority of pregnant women do not meet the recommended amount of duration for daily PA [20]. Women at risk for GDM spend most of their time performing sedentary behaviors, despite a low prevalence of contraindications to exercise [21]. The prevalence of moderate and vigorous PA among obese pregnant women decreased from 4.8% in middle pregnancy to 3% in later pregnancy [22].

The factors related to PA in pregnant women include sociodemographic factors, pregnancy-related factors, objective environmental factors, physiological factors, psychological and cognitive factors, and social support [23–26]. Among these factors, the PA self-efficacy of pregnant women was identified as the key factor associated with insufficient PA [27, 28]. According to Bandura [29], self-efficacy refers to the belief in one’s capabilities to organize and execute the courses of action required to produce given attainments. A person with high self-efficacy is more willing to pursue an activity, despite difficulties, than a person with lower self-efficacy [30]. Researchers identified self-efficacy as a mediator to overcome PA barriers and increase PA [31, 32].

Dominant strategies to improve self-efficacy belief include direct successful experience, vicarious experiences, verbal persuasion, and emotional arousal [29]. Direct successful experience, including previous personal achievements and success, is the strongest influencing factor in self-efficacy. A vicarious experience can be defined as a person’s reinforcement of self-belief by watching a similar individual succeed in certain situations (i.e., “if they can do it, I can do it”). Verbal persuasion refers to guiding the individual to believe that they can succeed in specific situations through positive feedback and verbal clues. Emotional arousal refers to how an individual’s physiological state and their interpretation of that state can affect whether an experience is empowering or disempowering for them. To obtain a sense of self-efficacy, an individual can achieve a behavior successfully, observe others doing an assignment successfully, gain positive feedback about completing an assignment, or adjust their physiological and psychological status [30]. PA interventions based on self-efficacy theory have previously had a positive effect on PA among heart failure patients [33] and overweight and obese women [34].

Theory-based interventions guided by the constructs of theory have been found to have more effective and long-term effects than those without a theory as a basis [35]. Using theories also allows for structured planning, implementation, and evaluation of the intervention, which enhances the effectiveness of the program [36]. Although some theories have been used to promote PA during pregnancy [27], the effectiveness of self-efficacy-oriented PA intervention among women with high-risk factors for GDM remains understudied.

In mainland China, women are expected to be seen during pregnancy every 4 weeks until 28 weeks’ gestation, every 2 weeks until 36 weeks, and then weekly until giving birth. It is estimated that a woman “booking” at 6–13^+6^ weeks’ gestation and delivering at 40 weeks makes 11 antenatal care visits [37]. However, health education on PA is not covered by routine antenatal care. Furthermore, no strong evidence currently exists regarding the best intervention for the prevention of GDM. In addition, most PA interventions for pregnant women are designed for healthy pregnant women or overweight pregnant women rather than women with multiple risk factors [13]. The evidence on the effectiveness of PA interventions for women with high-risk factors for GDM is still inconclusive [38–41]. Moreover, most study participants in the work designing these interventions were Caucasian women, although Wang et al. [38] conducted a randomized control trial of overweight and obese pregnant women in Beijing where participants in the intervention group engaged in a hospital-supervised cycling program (3 times/week) for the period of 24 ± 2 weeks. Even though the incidence of GDM was lower in their intervention group, this result can be difficult to achieve on a large scale as it may take a lot of equipment, professionals, time, and money.

A pragmatic parallel, randomized controlled trial was designed to investigate the effect of a self-efficacy-enhancing PA intervention on the incidence of GDM, blood sugar values, and PA level. The secondary objectives of this study are to investigate the effect of the PA program on PA self-efficacy; gestational weight gain; maternal outcomes, such as gestational hypertension, preeclampsia, and mode of delivery; and neonatal outcomes, including gestational age at delivery, preterm birth, Apgar score, birth weight, birth length, macrosomia, low birth weight, fetal distress, amniotic fluid contamination, number of large for gestational age (LGA) infants, and number of small for gestational age (SGA) infants.

## Methods/design

### Study design

This is a parallel, randomized controlled clinical trial that will be conducted in Zhengzhou, China. Participants attending the antenatal clinic of the study hospital will be randomly allocated to a study group receiving a self-efficacy-enhancing PA intervention or a control group receiving the usual care. Baseline assessment will occur before 12^+6^ gestational weeks. Follow-up measurements will occur at 24–28 weeks, 35–37 weeks, and within 3 days after delivery. Table 1 summarizes the study design and timeline. This study is supported by a research grant (grant no. 72174216) from the National Natural Science Foundation of China.

**Table 1:**
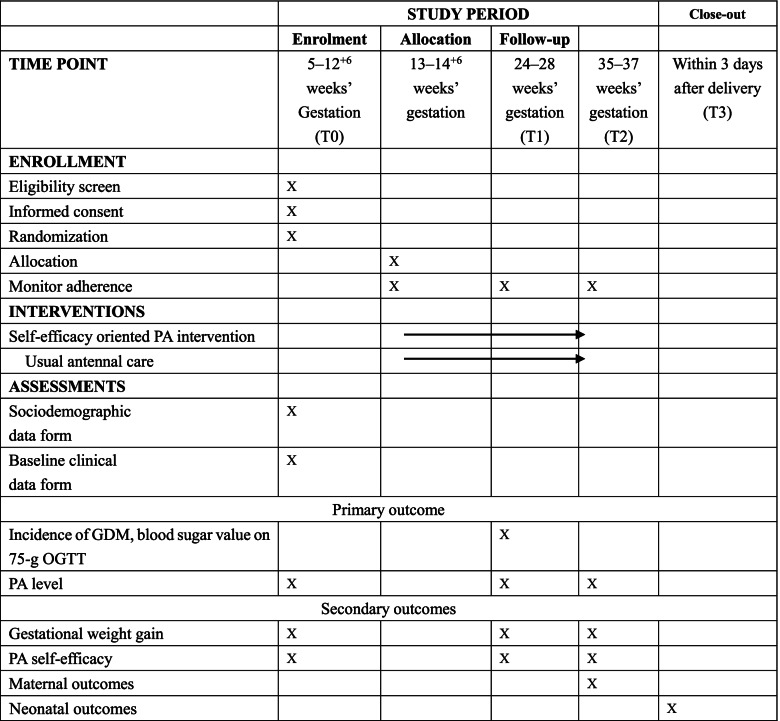
Study design and timeline

*Abbreviations*: *GDM* Gestational diabetes mellitus, *OGTT* Oral glucose tolerance test, *PA* Physical activity

The study protocol was designed using the Standard Protocol Items: Recommendations for Interventional Trials (SPIRIT) guidelines for interventional trials [42]. Any changes that need to be made to the trial protocol will be communicated to all investigators, the ethics committees, and the trial registry. The study has been approved by the Institutional Review Board of the School of Nursing at Sun Yat-sen University and by the study hospital.

### Eligibility criteria

Women who meet the following study inclusion criteria will be enrolled: (1) singleton pregnancy; (2) 5–12^+6^ weeks pregnancy; (3) ≥1 risk factor for GDM (e.g., maternal age ≥ 35 years old, pre-pregnancy BMI ≥ 24 kg/m^2^, family history of type 2 diabetes, polycystic ovary syndrome, previous macrosomia, previous GDM, previous glucose intolerance, previous fetal anomaly or hydramnion, current pregnancy with fetus growing greater than the gestational age, hydramnion, or repeated colitis).

Women will be excluded from this study if they have an exercise contraindication [18], such as severe cardiac or respiratory diseases, severe preeclampsia, uncontrolled hypertension, thyroid disease, type 1 diabetes, cervical insufficiency, persistent vaginal bleeding, threatened prematurity, placenta previa, ruptured membranes, poorly controlled anemia, fetal growth restriction, multiple pregnancies (≥ 3), bone or joint problems, severe obesity (body mass index > 35 kg/m^2^); are participating in other antenatal PA programs; or are currently being treated with metformin or corticosteroids.

### Sample size

The sample size of this study was calculated based on recent research available on the impact of PA on the incidence of GDM in Chinese overweight and obese pregnant women. Using information from that study [38] documenting incidence rates of GDM of 22% in the intervention group after PA intervention and 40.6% in the control group, we found that a sample size of a ≥ 98 women in each arm of this study will be required to detect a difference in the incidence of GDM between groups (80% power and *α* = 0.05). The treatment and control ratio is 1:1. Allowing for 20% attrition during pregnancy, ≥ 122 women in each arm will need to be recruited.

### Recruitment, setting, and informed consent

The present study will be undertaken in Zhengzhou, China, which is the capital of Henan Province and located in the central plains of China. Zhengzhou is a sub-provincial city and has a population of approximately 13 million people. The participants will be recruited from an antenatal clinic by researchers in a regional teaching hospital, which has > 6000 births annually. All pregnant women are required to visit the outpatient service to book into the hospital at their first clinical visit. Women will be recruited at the time of booking into the hospital at < 12^+6^ weeks’ gestation. The women who meet the eligibility criteria will be enrolled after they read and sign an informed consent form.

### Randomization and blinding

Pregnant women meeting the inclusion criteria and who agree to participate in our randomized controlled trial will be allocated randomly into either the intervention group or control group. Computer-generated randomization sequences will be provided by a biostatistician with no clinical involvement in the trial and stored securely on a password-protected computer. The randomization process will be done in blocks of four women. Each block will therefore result in the allocation of two women for the intervention and two women for the control group, ensuring a recruitment balance of 1:1 throughout the study.

Due to the nature of the intervention, it is not possible for study participants or the first researcher (Y. X.), who is delivering the intervention, to be blinded to the group allocations in this study. However, the other care providers, including nurses, midwives, doctors, and coaches, will be blinded to the group allocations. The first researcher (Y. X.) will assign women to the intervention or control group using a centralized, remote computer-generated randomizer called Sealed Envelope [43]. To minimize the risk of detection bias, every woman will be issued a unique three-digit study code. Therefore, the first researcher (Y. X.) will be blinded to the participant’s responses using the study code during data entry. Additionally, the first researcher (Y. X.) will be kept blinded to the group allocations during data analysis, and the statistician will analyze with the first researcher (Y. X.). All data, including both electronic and hard copies, will be destroyed 5 years after completion of the study.

### Treatment and assessment schedule (participant timeline)

**Fig. 1:**
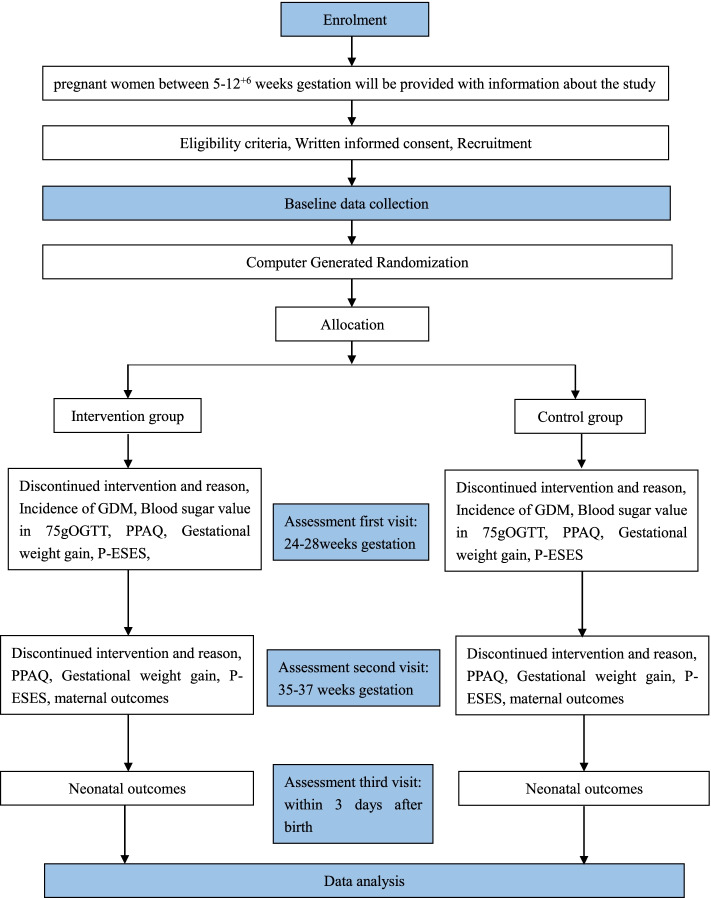
Pragmatic randomized controlled trial flowchart

The study period will run from approximately 5 weeks’ gestation until 3 days after birth. The duration of the self-efficacy theory-oriented antenatal PA program will be from approximately 13 to 14^+6^ weeks’ gestation until 35–37 weeks’ gestation. To assess the comparability of the study groups, sociodemographic and baseline clinical data will be obtained from the hospital database and pregnant women. The first follow-up will be conducted at 24–27^+6^ weeks’ gestation, and the second and third follow-ups will be performed at 35–37 weeks’ gestation and within 3 days after giving birth. Figure 1 outlines the process of the study.

### Intervention

Bandura’s self-efficacy theory [29, 30] guided the development of our self-efficacy-enhancing PA intervention to increase PA self-efficacy in women with high-risk factors for gestational diabetes mellitus; to decrease their incidence of GDM and blood sugar values; and to improve their PA level, proper gestational weight gain, maternal outcomes, and neonatal outcomes.

The intervention will start between 13 and 14^+6^ week’ gestation and will last for 24 weeks. After recruitment, about 8–10 pregnant women with similar expected dates of confinement will be included into a small group. The intervention will be conducted with the combination of face-to-face and online methods in small groups. The self-efficacy-enhancing intervention is complex.

The intervention includes the following components: (1) one 40-min face-to-face education session regarding GDM, healthy pregnancy weight gain, and PA during pregnancy conducted by an obstetric nurse and midwife; (2) one 50-min face-to-face exercise clinic visit conducted by a coach, obstetrician, nurse and midwife; (3) one 40-min daily exercise video including aerobic, resistance, and stretching exercises delivered to the women through WeChat platform (Tencent, Shenzhen, China); (4) daily reminders via the WeChat group to encourage the women to follow the 40-min video, performing the exercises and recording in their exercise diary; (5) one 40-min face-to-face education session regarding self-management of pregnancy-related symptoms conducted by an obstetric nurse and midwife; (6) one 40-min online education session regarding pregnancy-related emotion management conducted by an obstetric nurse, midwife and psychological consultant via Tencent Meeting (Tencent, Shenzhen, China); and (7) six offline and five online group discussion follow-up sessions (30–40 min for each session) every 2 weeks via Tencent Meeting or WeChat delivered by an obstetric nurse and midwife.

The initial face-to-face intervention will be delivered at the antenatal clinic of the study hospital during 13–14^+6^ weeks’ gestation. This first education session will focus on providing knowledge regarding the necessity and security of exercise during pregnancy. A booklet with the knowledge will be provided to participants. After the education session, the coach will conduct a face-to-face group exercise clinic visit to teach the pregnant women how to exercise safely under the supervision of obstetricians, nurses, and midwives. The coach will discuss the steps participants can take to help them begin exercising. For example, participants could ask their friends and family members to join them in activities like brisk walking instead of more sedentary activities, or begin by completing 10 min of moderate PA daily and increasing this amount gradually to 30 min. Additionally, the coach will guide the participants to exercise together following the exercise video.

The exercise video lasts for 40 min, including a 5-min warm-up, 30-min main exercise section, and a 5-min cool-down. The movements in the exercise video have been recommended by the coach. The warm-up and cool-down sessions contain stretching exercises for the arms and legs at a light intensity. The main section includes aerobic exercises (walking briskly) and resistance (bodyweight) training at a moderate intensity. Aerobic and resistance exercises will be repeated 10–15 times for each movement. The moderate intensity of the exercises will be set according to each woman’s perceived effort (within the range of 12–14 points on the Borg Scale) [44] and Talk Test (one can talk but not sing during exercise) [45]. Participants can adjust the exercise intensity and frequency according to the progress of their pregnancy.

The exercise video will be uploaded to the WeChat platform, a free application released in 2011 by Tencent. With its convenience and various services, including instant messaging, free phone calls, group discussion, sharing moments, mobile payment, small programs, and public accounts, WeChat has become the most prevalent social networking platform in China. The latest data from the 2020 Social Media User Behavior in China report indicates that 88.1% of WeChat users open their WeChat app every day, including 53.9% who do so for > 2 h per day [46]. We will advise the women to exercise at home following the exercise video for ≥ 5 days a week. Researchers will remind the women to exercise and record exercise diaries every day through the WeChat group.

An offline group discussion at the antenatal clinic of the study hospital or online group discussion via Tencent Meeting will be delivered by an obstetric nurse and midwife every 2 weeks. These sessions will aim to strengthen participants’ self-efficacy in PA and will be guided by a protocol. Pregnant women in small groups will sit around to discuss the problems that arise when doing exercise and share solutions on how to keep active with each other. Researchers will analyze the group members’ gestational weight gain and PA status in the prior 2 weeks. Feedback will be given to every group member. The nurse will provide encouragement and reinforcement of the participants’ efforts and successes and will empower them through support. Successful pregnant women will be invited to share their self-management strategies as role models. The duration of each group discussion session will vary from 30 to 40 min according to group members’ needs.

During 15–16^+6^ weeks’ gestation, face-to-face knowledge education about pregnancy-related symptom management will be provided by obstetric nurses and midwife at the antenatal clinic of the study hospital. After that, an offline group discussion will be held at the antenatal clinic of the study hospital. During 17–18^+6^ weeks’ gestation, online knowledge education about pregnancy-related emotion management will be offered by an obstetric nurse, midwife and psychological consultant online via Tencent Meeting. A group discussion will be held online after the knowledge education session by an obstetric nurse.

The four strategies recommended by Bandura [29] were incorporated into each component of the intervention. Accomplishment experiences include identifying potential barriers and discussing self-management strategies for being physically active every day, goal-setting, the use of the exercise video to practice at home, and the booklet for reinforcing knowledge. Vicarious experiences include participants who had successfully done exercise according to the coach’s guidance sharing their self-management skills to keep active. Verbal persuasion includes providing encouragement and acknowledging participants’ ability to keep themself active. Physiological monitoring includes the explanation for why symptoms and negative emotions might occur during pregnancy, and the discussion of management strategies. Table 2 outlines examples of the four strategies [29].

**Table 2:** Strategies used in the self-efficacy-enhancing intervention

Performance accomplishments	Identifying the obstacles to keeping participants active through discussion
	Setting achievable goals and actions, e.g., achieving 10 min of exercise following the exercise video daily before increasing gradually to 30 min
	Negotiating techniques with participants to achieve bigger goals, e.g., set alarm on phone for activity; put notes on doors, the refrigerator, or the television to be active; stand or walk rather than sitting in add breaks
	Monitoring physical activity diary and gestational weight gain on WeChat notes
	Planning for decreasing sedentary behavior
	Providing positive feedback for participants’ accomplishments
	Providing booklet to reinforce knowledge
Vicarious experience	Checking behavioral tracking, review, and feedback on goals; “we’re going to check how you went with your physical activity and tracking and work together to set a healthy activity goal.”
	Sharing self-management strategies from successful pregnant women
Verbal persuasion	Discussing and providing information about consequences of physical inactivity and unhealthy gestational weight gain
	Confirming participants have the capability for exercise and weight self-management
	Informing that one’s own behavior may be an example to others, i.e., inform the participants that if they do physical activity, that may be a good example for their friends and family members.
	Guiding participants to recall previous successful behavior-change situations, discuss context and factors associated with success
	Providing positive feedback for the participant’s effort
Physiological states	Assessing and explaining the participant’s pregnancy-related symptoms and negative emotions
	Discussing strategies for managing symptoms, anxiety, or depression, such as positive self-talk and muscle relaxation

The intervention will be conducted by a multi-disciplinary team of obstetricians, nurses, midwives, psychological consultant, and coaches, all of whom have had >10 years of experience in their professional field. A 4-h training session will be provided to the team members by the first researcher (Y. X.) before the study begins. The study protocol will be reviewed and discussed to ensure consistency in delivering the PA intervention.

The Medical Research Council guidelines for the evaluation of complex interventions will be used to explore the process by which the intervention may have led to its effect or not [47]. Multiple methods of data collection will be used to assess the implementation of the intervention, the mechanisms of impact, and context effects (Table 3).

**Table 3:** Process evaluation items, indicators, and methods of measurement

Process evaluation items		Indicators	Measurement
**Implementation**			
Dose	Quantity of the intervention implemented	Number of sessions received Frequency and duration of exercising with the exercise video	Intervention logbook; WeChat record
Reach	How well the intervention is received	Percentage of pregnant women who met the inclusion criteria attend this study Percentage of participants who completed the intervention	Intervention logbook; WeChat record
Fidelity	Whether the intervention was delivered to participants according to the protocol	If the session was delivered to participants, gestational weeks, begin-time, complete-time; if the session was completed, reasons for participation and declining participation	Intervention logbook; WeChat record; semi-structured interviews
Adaptation	Extent to which adaptation across contents is acceptable	Satisfactory rate	Satisfactory questionnaire; Semi-structured interviews
**Mechanism of impact**			
Participant responses	Participant responses to and interactions with the intervention	How satisfied are participants with the content, work procedures, and delivery? What is their opinion of the physical activity program’s appropriateness/usefulness, acceptability, feasibility, sustainability?	Satisfactory questionnaire; Semi-structured interviews
**Context**	Which factors/circumstances have either facilitated or hindered working with the interventions?	Which factors/circumstances have either facilitated or hindered working with physical activity programs?	Semi-structured interviews.

Adherence to the PA intervention will be strongly emphasized and registered in the women’s training diaries and reports from the individuals leading the training groups. Exercise participants who develop obstetric contraindications to exercise [18] will discontinue the exercise intervention but be included in the intention-to-treat analysis.

### Control group

Participants in the control group will receive the customary regular consultations with the midwife, nurse, or obstetrician. They will not be discouraged from exercising on their own. Neither group will receive special recommendations about diet or other standard antenatal care delivered through prenatal health education.

### Outcome measures and data collection

The list of data-collection instruments and times of data collection are presented in Table 4. The primary outcomes of this study are blood sugar values during the 75-g oral glucose tolerance test, the incidence of GDM at 24–28 weeks’ gestation, and PA levels at 24–28 and 35–37 weeks’ gestation. The secondary outcomes include PA self-efficacy, gestational weight gain, maternal outcomes, and childbirth outcomes.

**Table 4:** Data collection instruments and time of data collection

	Booking		24–28 weeks’ gestation		35-37 weeks’ gestation		Within 3 days after delivery	
	IG	CG	IG	CG	IG	CG	IG	CG
**Primary outcomes**								
Blood sugar value on 75-g OGTT (medical database); incidence of GDM			√	√				
PA level (PPAQ)	√	√	√	√	√	√		
**Secondary outcomes**								
PA self-efficacy (P-ESES)	√	√	√	√	√	√		
Gestational weight gain (questionnaire)	√	√	√	√	√	√		
Maternal outcomes (medical records)					√	√		
Neonatal outcomes (medical records)							√	√

*Abbreviations*: *CG* Control group, *GDM* Gestational diabetes mellitus, *IG* Intervention group, *OGTT* Oral glucose tolerance test, *P-ESES* Pregnancy Exercise Self-efficacy Scale, *PA* Physical activity, *PPAQ* Pregnancy Physical Activity Questionnaire

According to the guidelines for the management of gestational diabetes mellitus in China [48], pregnant women with a fasting glucose level of ≥ 7.0 mmol/L or random blood glucose level of ≥ 11.0 mmol/L in early pregnancy are diagnosed with pre-gestational diabetes mellitus. Women who are not diagnosed with pre-gestational diabetes mellitus will receive a 75-g oral glucose tolerance test at 24–28 weeks’ gestation. Using the criteria of the International Association of Diabetes and Pregnancy Study Groups, GDM will be diagnosed when the fasting plasma glucose value is greater than the following standards: 5.1 mmol/L, 10.0 mmol/L in 1 h, or 8.5 mmol/L in 2 h [49].

PA will be measured by the Pregnancy Physical Activity Questionnaire (PPAQ) [50]. Research has shown that PPAQ is reliable and currently the best-available tool to assess PA during pregnancy [51]. The original version of the PPAQ contains 32 items to assess household/caregiving activities (13 items), occupational activities (5 items), sports/exercise activities (8 items), transportation activities (3 items), and physical inactivity (3 items) [50]. For each item, participants select a category of the amount of time spent carrying out the specific activity ranging from 0 to ≥ 6 or ≥3 h/day or 0 to ≥ 3 h/week. According to the calculation guideline of PPAQ [50], each item refers to a given metabolic equivalent of task (MET) value, which describes the PA levels measured in metabolic equivalents. Subsequently, the amount of time spent when carrying out each activity is converted into the MET value. According to the MET value, the total 32 activities could be divided into sitting (< 1.5 METs), low-intensity activity (1.5–2.9 METs), moderate-intensity activity (3.0–6.0 METs), and high-intensity activity (> 6.0 METs). The Chinese version of the PPAQ contains 31 items after deleting the item of “holding babies” [52]. The Chinese version of the PPAQ has been demonstrated to have good psychometric properties, with a content validity of 0.94 and test–retest reliability of 0.94.

PA self-efficacy will be measured by the Pregnancy Exercise Self-efficacy Scale (P-ESES) [53], a 10-item instrument where each item is rated on a 5-point Likert scale ranging from 5 points (strongly agree) to 1 point (strongly disagree). Higher scores indicate a greater level of PA self-efficacy. According to the total score of P-ESES, PA self-efficacy will be divided into a high level (41–50 points), medium level (21–40 points), and low level (10–20 points). The Chinese version of P-ESES contains three subscales: four items in the overcome exercise barriers subscale, two items in the overcome emotion barriers subscale, and four items in the overcome support barriers subscale [54]. The Chinese version of P-ESES has been demonstrated to have good psychometric properties, with a Cronbach’s *α* value of 0.80 and test–retest reliability of 0.53.

Gestational weight gain (GWG) will be calculated as the difference between weight measured at the time of inclusion, 24–28 weeks’ gestation, and 35-37 weeks’ gestation. pre-gestational BMI will be calculated using the self-reported pre-pregnancy weight and height [weight (kg)/(height (m))^2^]. According to the Working Group on Obesity in China [55], individuals may be classified as underweight (BMI < 18.5 kg/m^2^), normal weight (BMI 18.5–23.9 kg/m^2^), overweight (BMI 24.0–27.9 kg/m^2^), or obese (BMI ≥ 28 kg/m^2^) according to their BMI. We will measure maternal body weight to the nearest 0.1 kg with a calibrated electronic scale (RGZ-50; SUHONG Medical Instrument, Jiangsu, China) with participants wearing indoor clothing and no shoes. Excessive gestational weight gain will be identified according to the 2009 guideline issued by the Institute of Medicine [56]. GWG will be categorized as inadequate, normal, or excessive GWG by the same guidelines [56]. Here, inadequate GWG will be defined as a bodyweight gain < 11.5 kg for normal-weight women, < 7 kg for overweight women, or < 5 kg for obese women; normal GWG will be defined as a bodyweight gain of 11.5–16 kg for normal-weight women, 7–11.5 kg for overweight women, or 5–9 kg for obese women; and excessive GWG will be defined as a bodyweight gain > 16 kg for normal-weight women, > 11.5 kg for overweight women, or > 9 kg for obese women, respectively.

Maternal outcomes will include gestational hypertension (defined as a blood-pressure elevation [systolic blood pressure > 140 mmHg or diastolic blood pressure > 90 mmHg] at > 20 weeks’ gestation in the absence of proteinuria) [57], preeclampsia (presence of both high blood pressure and proteinuria at 20 weeks’ gestation), premature rupture of membranes (rupture of membranes before 37 weeks’ gestation or at the onset of delivery), postpartum hemorrhage (blood loss > 500 mL within 24 h of delivery), and mode of delivery (vaginal, operative vaginal, or cesarean [elective or emergency]).

Neonatal outcomes will include gestational age at delivery, preterm birth (< 37 gestational weeks), Apgar score, birth weight, birth length, macrosomia (birthweight > 4000 g), low birth weight (birthweight < 2500 g), fetal distress (fetal hypoxia during pregnancy or labor), and amniotic fluid contamination (presence of meconium in the amniotic fluid), number of LGA infants (birthweight > 90th percentile for gestational age), and number of SGA infants (birthweight < 10th percentile for gestational age). Both LGA and SGA will be defined by international standards for sex-specific newborn size for each gestational age based on data from the Newborn Cross-sectional Study subpopulation [58].

### Data management

The first researcher (Y. X.) will be responsible for monitoring the collection and quality of data as well as the participant recruitment and retention. All data collected will be kept strictly confidential, accessed only by members of the trial team, and stored on a secure computer with passwords. Each participant will be allocated an individual trial identification number.

### Statistical analysis

All data will be analyzed by SPSS version 26 software (IBM Corporation, Armonk, NY, USA). Descriptive statistics, such as frequencies and percentages of categorical variables and mean, standard deviation, median, and range values of linear variables, will be used. To gain insight into the relative factors of PA, descriptive statistics will be calculated to describe participants’ demographic characteristics, gestational weight gain, PA level, and PA self-efficacy during pregnancy. Primary analyses, including chi-squared and independent *t* tests, will be used to examine group differences in the primary and secondary outcomes. Repeated analysis of variance will be used to assess within-patient correlations. By using a series of mixed-effects regression models with a likelihood-based approach, we will regress the score of PPAQ at follow-up to determine the effect on the baseline, group, time, group × time, and confounders identified in the preliminary step. Effect sizes will be reported with 95% confidence intervals, and results will be considered significant if *P* < 0.05. The analysis of the primary and secondary outcomes will be based on an intention-to-treat basis [59], which will include study withdrawals and losses to follow-up.

## Discussion

PA throughout pregnancy has been proven safe and beneficial for both women and fetuses. However, women at higher risk for GDM spent most of their time performing sedentary behaviors rather than PAs [21]. Although researchers have identified self-efficacy as a mediator to overcome PA barriers [31, 32], exercise intervention during pregnancy based on self-efficacy theory has not been discussed so far. Furthermore, there is conflicting evidence regarding the positive effect of exercise in women at higher risk for GDM on the incidence of GDM and other maternal or neonatal outcomes. With the development of China's second-child and third-child policies, there will be more and more pregnant women with complex high-risk factors for GDM. Our proposed randomized controlled trial will provide important additional information on the effect of self-efficacy theory-oriented exercise programs on pregnant women with multiple high-risk factors for GDM.

This study has several strengths. First, it targets pregnant women at high risk for GDM. Other strengths of our study include the development of intervention sessions based on self-efficacy theory; the thorough testing of pregnant women as well as their newborns; and the investigation of possible effects of exercise training on PA level, the incidence of GDM, and gestational weight gain. In addition, the composition of the exercise program includes both aerobic and resistance training. Because of the convenience and ease of access, WeChat was selected as the delivery channel for the exercise video and exercise diary. Moreover, we conducted a process evaluation following the guidance of Medical Research Council guidelines.

The limitation of this study is that the PA level will be evaluated by a questionnaire without objective equipment. Guidelines about PA for pregnant women recommend subjective assessments as the first choice to evaluate exercise status as they are handy and personalized [18]. We will ask our study participants to keep an exercise diary, including comments on walk steps and their feelings while exercising. The data above will be involved in the process evaluation. Primary risks at the participant level will include failure to persist with PA with standard posture for the movements or forgetting to exercise. To mitigate against these risks, participants will receive training regarding how to follow the exercise video at home and how to complete their exercise diary on the WeChat platform. We will also offer some tips to remind them to exercise and record their experience, such as setting a clock alarm, putting notes on the refrigerator or television to reduce sedentary time, and being active. Moreover, researchers will send reminder messages every morning or evening to the WeChat group to remind participants to exercise.

According to the World Health Organization [60], some PA are better than none for pregnant women. The results from this study will provide grounds for giving advice as well as organizing exercise training groups for women with high-risk factors for GDM. If the women randomized to the exercise program achieve positive effects in any primary or secondary outcome compared to controls, the addition of this program to the regular pregnancy care for high-risk individuals should be considered.

## Conclusion

This paper describes how we plan to implement a theory-based PA program during pregnancy to reduce the incidence of GDM and improve the PA level, PA self-efficacy, and gestational weight gain. We will also conduct a process evaluation. The effectiveness of the proposed program will be investigated in the future in a randomized controlled trial. The results will speak to whether a low-touch, mobile technology intervention can effectively reduce the incidence of GDM and improve PA, PA self-efficacy, and gestational weight gain in pregnant women suffering at high risk for GDM. The results could inform the development and implementation of broadly reaching interventions in the community, academic, and clinical settings and could thus play an important role in the effort to improve the health of pregnant women and newborns.

## Trial status

This study is approved by the Chinese Clinical Trial Registry (CHiCTR): (protocol ChiCTR2200056355). Recruitment commenced in February 2022. It is expected to be completed by October 2022. Data collection will be completed by February 2024.

## Data Availability

The datasets analyzed during the current study are available from the corresponding author on reasonable request.

## Abbreviations

BMI: Body mass index
GDM: Gestational diabetes mellitus
GWG: Gestational weight gain
LGA: Large-for-gestational-age
MET: Metabolic equivalent of task
PA: Physical activity
P-ESES: Pregnancy Exercise Self-efficacy Scale
PPAQ: Pregnancy Physical Activity Questionnaire
SGA: Small for gestational age
